# Imaging findings for pancreatic Hamartoma: two case reports and a review of the literature

**DOI:** 10.1186/s12876-020-1185-8

**Published:** 2020-02-17

**Authors:** Heng Cui, Yuqing Lian, Feng Chen

**Affiliations:** Department of Radiology, the First Affiliated Hospital, College of Medicine, Zhejiang University, 79 Qingchun Road, Hangzhou, 310003 Zhejiang China

**Keywords:** Imagine, Pancreas, Hamartoma, Benign

## Abstract

**Background:**

Pancreatic hamartoma is an extremely rare benign disease, and previous reports have provided little detail regarding its appearance in imaging. As a result, we report the imaging findings for two cases of pancreatic hamartoma.

**Case presentation:**

One 57-year-old female patient and one 69-year-old male patient presented with pancreatic lesions incidentally detected by US; CT and MRI revealed a 2.9-cm cystic and solid lesion and a 1.4-cm solid lesion, respectively. US showed a hypoechoic well-defined mass in the pancreatic head. The plain CT indicated that the internal density was uneven, and the lesions showed obvious progressive enhancement. The MRI-T2WI showed iso- to high-intensity, the DWI showed iso-intensity, and the masses also all showed obvious progressive enhancement. Histopathological studies confirmed the diagnosis of pancreatic hamartoma.

**Conclusion:**

Pancreatic hamartoma is an extremely rare tumour with benign features, such as no dilatation of the MPD and well-defined, slight hyperintensity or iso-intensity on T2WI and iso-intensity on DWI, with obvious progressive enhancement. Therefore, detailed review of multiple imaging modalities may help in diagnosis of PH and prevent unnecessary surgery for patients with this diagnosis.

## Background

Pancreatic hamartoma (PH) is an extremely rare and benign disease of the pancreas that account for approximately 10% of pancreatic primary mesenchymal tumours [1]. At present, only 37 cases have been reported in the literature [2–24]. PH is easily misdiagnosed as other tumours, including malignant tumours, which has great effects on patients. Knowledge of the imaging characteristics of this disease is very important. Previous reports have mostly focused on the clinical and pathological features of PH and have provided less detail regarding its imaging appearance. In this paper, we report the imaging characteristics observed for two cases of PH, including ultrasonography (US), computed tomography (CT) and magnetic resonance imaging (MRI) features, and retrospectively review the imaging findings for previously reported cases.

## Case presentation

### Case 1

**Fig. 1:**
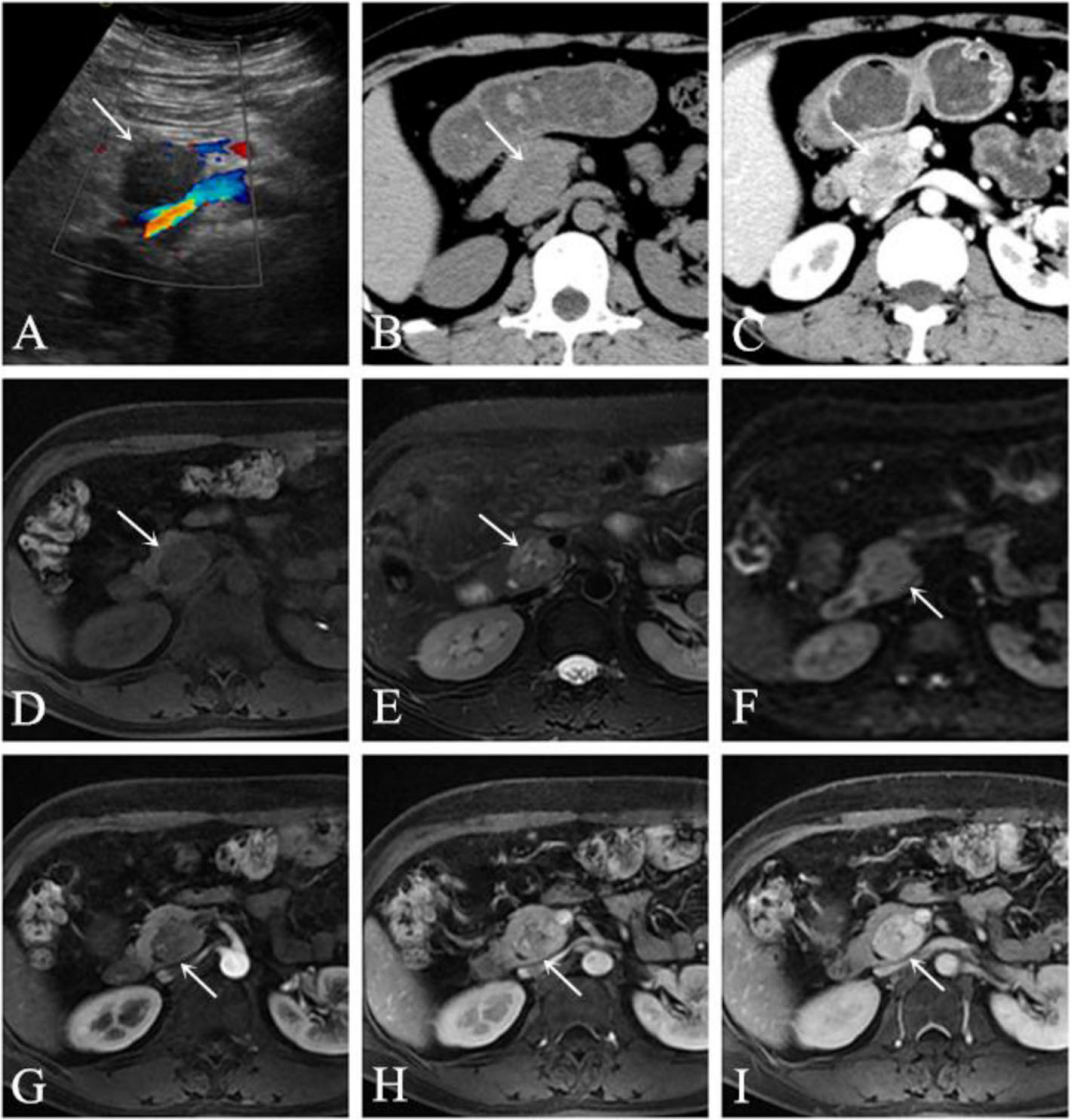
The lesion showed a hypoechoic mass in the pancreatic head on US (**a**). Axial CT of the abdomen (plain scan and venous phase) showing a 2.9-cm iso-density lesion in the pancreatic head that was well-defined after enhancement (**b-c**). Axial pancreatic MRI showing a solid lesion with several small cystic lesions, T1WI showed hypointensity (**d**), T2WI showed iso- to high-intensity (**e**), and DWI showed iso-intensity (F); the lesion had obvious progressive enhancement (**g-i**)

A 57-year-old female patient was admitted to our hospital due to the discovery of a pancreatic space-occupying lesion 1 week prior. The patient had hypertension for more than 10 years that was controlled by reserpine. Laboratory testing showed that the serum levels of amylase, bilirubin, and tumour markers were not elevated. US showed a hypoechoic mass in the pancreatic head that had no obvious blood echoes; the boundary was clear. Contrast-enhanced CT showed that the lesion was approximately 2.9 × 2.3 cm^2^ in size and that the internal density was uneven; the lesion showed obvious progressive enhancement. The boundary was clear after enhancement. The unenhanced CT value was approximately 27–68 Hounsfield units (HU). Contrast-enhanced CT scanning showed a focus with inhomogeneous enhancement, and the CT value was approximately 24–85 HU in the arterial phase and 66–155 HU in the venous phase. The MRI indicated a space-occupying lesion on the uncinate process of the pancreas; the shape was regular, the boundary was clear, the internal intensity was slightly uneven, and there were several small cystic lesions. The T1-weighted image (T1WI) showed low-intensity, the T2-weighted image (T2WI) showed iso- to high-intensity, the diffusion-weighted image (DWI) showed iso-intensity, and the mass showed obvious progressive enhancement (Fig. 1). There was no dilation of the main pancreatic duct, no evidence of invasion of the surrounding organs, and no retroperitoneal lymphadenopathy. The pancreatic neuroendocrine tumour was initially diagnosed. As the patient had the desire for surgery, a Whipple resection was performed after the surgical evaluation. The histopathological studies confirmed the diagnosis was PH. So far, the patient has been followed up for 34 months and has remained disease free.

### Case 2

**Fig. 2:**
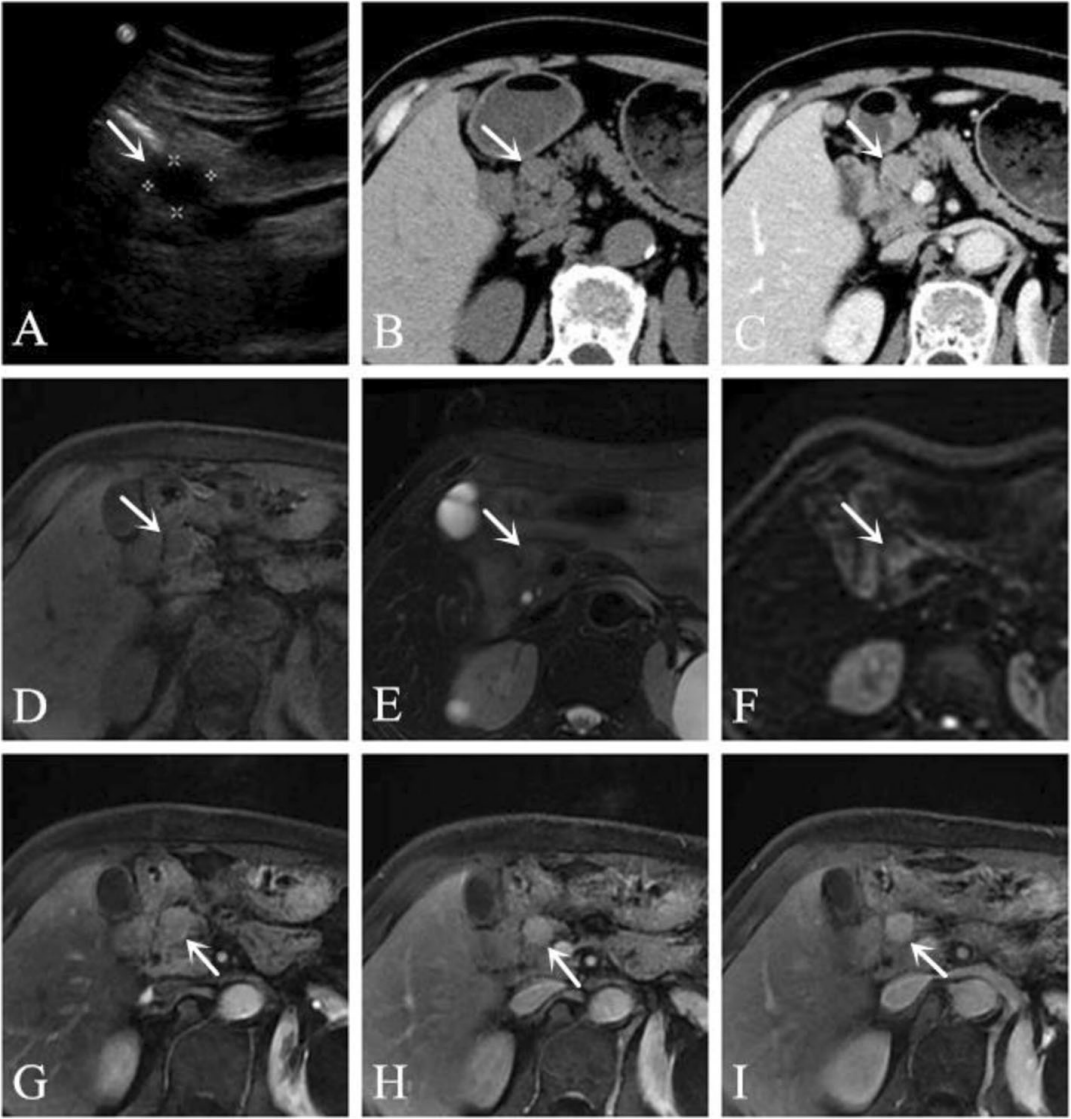
The lesion showed a hypoechoic mass in the pancreatic head on US (**a**). Axial CT of the abdomen (plain scan and venous phase) showing a 1.4-cm iso-density lesion in the pancreatic head that was well-defined after enhancement (**b-c**). Axial pancreatic MRI showing a solid lesion, T1WI showed hypointensity (**d**), T2WI showed iso-intensity (**e**), DWI showed iso-intensity (**f**), the lesion had obvious progressive enhancement (**g-i**)

A 69-year-old male patient was discovered to have had a pancreatic space-occupying lesion for 1 month during a medical examination. The patient gave no medical history of diseases of the pancreas. The neoplasm markers were within the normal range, and pancreatic exocrine and endocrine function were normal. US showed a hypoechoic mass in the pancreatic head. CT of the abdomen displayed a lesion that was approximately 1.4 × 1.2 cm^2^ in size, and the lesion showed obvious progressive enhancement. The unenhanced CT value was approximately 33–42 HU. Contrast-enhanced CT scanning showed inhomogeneous enhancement with a CT value of 45–60 HU in the arterial phase and 68–80 HU in the venous phase. MRI indicated a space occupying lesion in the head of the pancreas; the shape was regular, the boundary was clear, and the internal intensity was slight uneven. T2WI and DWI showed iso-intensity, and the mass showed obvious progressive enhancement (Fig. 2). As it was difficult to determine whether the tumour was malignant, the patient underwent a Whipple resection. The final pathological diagnosis was PH. So far, the patient has been followed up for 44 months and has remained disease free.

### Findings of operation and pathology

**Fig. 3:**
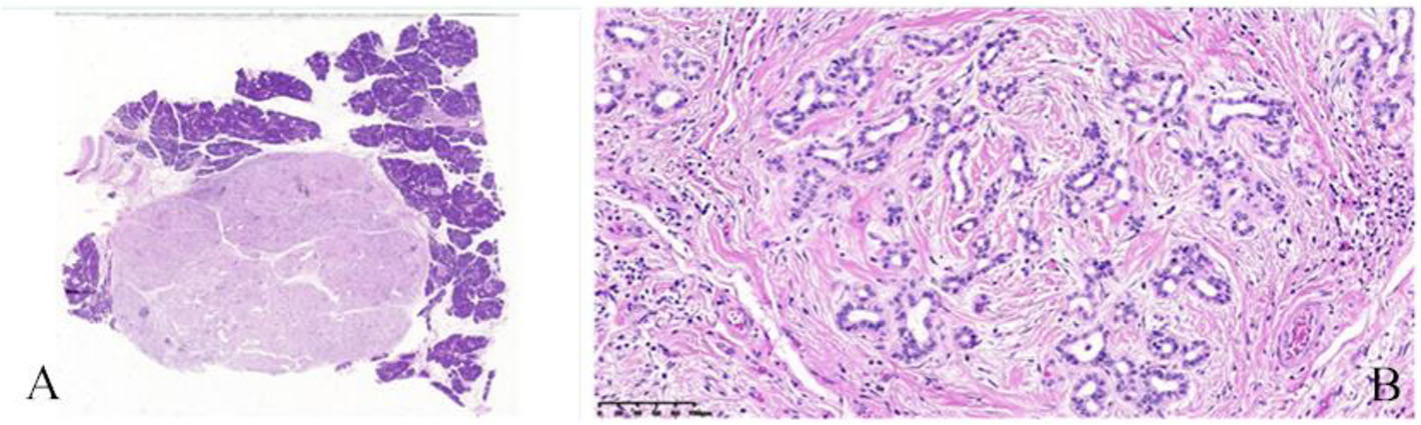
Microscopic examination with low power (×10) (**a**) and high power (×100) (**b**) revealed a relatively well-demarcated mass without capsule (**a**). The lesion consisted of haphazardly arranged normal acini, well-differentiated small glandular ducts and obvious interstitial fibrosis (**b**)

In the operation, one tumor was located in the pancreatic uncus with the diameter 2.5-cm, and the other in the head of the pancreas with the diameter 1.5-cm, was slightly soft in texture, with good mobility. Both of the tumors had no capsules, with clear boundary. And no retroperitoneal lymph node metastasis was found. Microscopy (Fig. 3):a large number of well-differentiated small glandular tubules were clustered in the tumors, interstitial fibrosis was obvious, lymphocyte infiltration was observed, and the pancreatic parenchyma and glandular ducts were crossed. Immunohistochemistry results showed: CD138 (−), IGg4 (−), IGg (−), CK (pan) (−), Vimentin (+), Ki-67 (+), SMA (+).

## Discussion and conclusions

PH is an extremely rare benign disease of the pancreas. To the best of our knowledge, only 37 cases have been reported in the English literature. This disease may occur at any age. The youngest patient reported was a premature female infant who died of a trisomy 18 karyotype deformity after less than one hour of life [20], while the oldest patient reported was a 78-year-old woman [12]. The average age of diagnosis was approximately 51 years, and no gender predilection has been reported in the literature [2–24]. Several patients had concomitant abdominal or back discomfort, indigestion and weight loss [5–7, 10, 15, 19], while the majority of patients were asymptomatic. This benign tumour was often located in the head of the pancreas, and the rate was approximately 62% (23/37) [24]. Dilation of the main pancreatic duct (MPD) and bile duct or signs of jaundice were rare. Only three cases showed biliary or pancreatic duct obstruction [3, 17]. Consistent with the previous findings, our cases were diagnosed in elderly patients, occurred in the pancreatic head and were asymptomatic, with no dilation of duct. Most of these tumours were solitary, as only one case with three well-demarcated solid nodules in the head of the pancreas has been reported [12]. PH is usually divided into two subgroups: solid lesion types or solid and cystic lesion types, with no significant differences in the ratio (20/17). Case 1 was a solid lesion with small cystic lesions, and case 2 was a solid lesion. This tumour is characterized by soft tissue masses of different sizes (ranging from 0.3 to 14 cm), while over 50% of cases (29/37) were no more than 3 cm [6, 7, 9–13, 15–24]. On US, PH is usually observed as a hypoechoic mass with a clear boundary [11, 17, 19, 21]. On CT, the mass is usually well defined, and the internal density of most of the solid lesions is slightly uneven, with low iso-intensity and heterogeneous progressive contrast enhancement in the delayed phase [9, 13]. Additionally, the borders are typically well-defined after contrast enhancement [9, 22]. Similar to previous reports, our cases had heterogenous internal content, with obvious progressive enhancement. This observation is different from pancreatic adenocarcinoma and pancreatic neuroendocrine tumor. Pancreatic adenocarcinoma is characterized by low enhancement and surrounding structure invasion, and pancreatic neuroendocrine tumor by obvious enhancement at early phase after contrast administration. On MRI, the shape of PH lesions was often regular, and the boundary was clear on T2WI. The internal intensity was slightly uneven, T1WI showed low-intensity, T2WI showed iso- to high-intensity [12, 19, 21, 22], and DWI showed iso-intensity [21]. Similarly, our two cases showed iso-intensity on DWI, which is different from some kinds of pancreatic tumours with hyperintensity. For the pathological examination, there were many glandular ducts in the tumour, which might be related to the iso-intensity on DWI. The enhancement patterns were progressive and obvious enhancement in the portal phase or delayed phase was observed [9, 12, 13, 16, 22]. Our imaging features were consistent with those findings in the literature; therefore, it is suggested that the DWI and enhancement pattern are the most important features to indicate PH. On positron emission tomography (PET)-CT, some reports have shown that pancreatic hamartoma has normal 18F-fluorodeoxyglucose (FDG) accumulation [6, 12, 22]. However, Nagano’s research [21] showed lesion uptake of 18F-FDG with a maximum standardized uptake value (SUVmax) of 3.6 at early imaging (1 h), which increased to 5.0 on the delayed imaging, which made it difficult to differentiate pancreatic hamartoma from malignant tumours. In terms of the tumour capsule, there are still some controversies in the literature: some reports have shown the presence of capsules in PH [16, 18, 19, 24], while others have not [7, 9, 13, 22]. Unfortunately, the studies that reported tumour capsules did not show the imaging findings. Some reports showed that several cases had some lipomatous components [18, 24] or calcification [6], but this was very rare. PH is a benign tumour, and no recurrence has been reported. To date, the two patients presented here have been followed for 34 and 44 months, respectively, and have remained disease free. Even though PH is benign, it is difficult to diagnose via imaging. Most patients undergo surgical resection because of the possibility of malignant disease. Thus, useful information from imaging would help to select the best treatment.

Pancreatic hamartoma is an extremely rare and benign tumour of the pancreas. Clinical diagnosis based on imaging remains difficulty. We suggest that the observation of “well-demarcated lesion in the head of pancreas, with no dilatation of the MPD, slightly hyperintensity or iso-intensity on T2WI, iso-intensity on DWI, and obvious progressive enhancement patterns” may help in diagnosis of PH and prevent unnecessary surgery for patients with this diagnosis.

## Data Availability

The clinical and imaging data are available from the corresponding author upon request.

## Abbreviations

CT: Computed Tomography
DWI: Diffusion-Weighted Image
FDG: Fluorodeoxyglucose
HU: Hounsfield Unit
MRI: Magnetic Resonance Imaging
PET: Positron Emission Tomography
PH: Pancreatic Hamartoma
SUVmax: Maximum Standardized Uptake Value
T1WI: T1-Weighted Image
T2WI: T2-Weighted Image
US: Ultrasonography
